# The clinical impact of bacteremia on outcomes in elderly patients with pyelonephritis or urinary sepsis: A prospective multicenter study

**DOI:** 10.1371/journal.pone.0191066

**Published:** 2018-01-24

**Authors:** Arturo Artero, Luis Inglada, Ana Gómez-Belda, Josep A. Capdevila, Luis F. Diez, Alexandra Arca, José M. Romero, Marta Domínguez-Gil, Cristina Serra-Centelles, Javier de la Fuente

**Affiliations:** 1 Department of Internal Medicine, Hospital Universitario Dr. Peset. Universitat de València, València, Spain; 2 Department of Internal Medicine, Hospital Universitario Rio Hortega, Valladolid, Spain; 3 Department of Internal Medicine, Hospital Universitario Dr. Peset, Valencia, Spain; 4 Department of Internal Medicine, Hospital de Mataró, Barcelona, Spain; 5 Department of Internal Medicine, Hospital Torrecárdenas, Almería, Spain; 6 Department of Internal Medicine, Hospital Povisa, Vigo, Spain; 7 Department of Microbiology, Hospital Universitario Rio Hortega, Valladolid, Spain; Duke University School of Medicine, UNITED STATES

## Abstract

**Background:**

Bacteremia is common in severe urinary infections, but its influence on the outcomes is not well established. The aim of this study was to assess the association of bacteremia with outcomes in elderly patients admitted to hospital with pyelonephritis or urinary sepsis.

**Methods:**

This prospective muticenter observational study was conducted at 5 Spanish hospitals. All patients aged >65 years with pyelonephritis or urinary sepsis admitted to the departments of internal medicine and with urine and blood cultures obtained at admission to hospital were eligible. Transfer to ICU, length of hospital stay, hospital mortality and all cause 30-day mortality in bacteremic and non-bacteremic groups were compared. Risk factors for all cause 30-day mortality was also estimated.

**Results:**

Of the 424 patients included in the study 181 (42.7%) had bacteremia. Neither transfer to ICU (4.4% vs. 2.9%, p = 0.400), nor length of hospital stay (9.7±4.6 days vs. 9.0±7.3 days, p = 0.252), nor hospital mortality (3.3% vs. 6.2%, p = 0.187), nor all cause 30-day mortality (9.4% vs. 13.2%, p = 0.223) were different between bacteremic and non-bacteremic groups. By multivariate analysis, risk factors for all cause 30-day mortality were age (OR 1.05, 95% CI 1.00–1.10), McCabe index ≥2 (OR 10.47, 95% CI 2.96–37.04) and septic shock (OR 8.56, 95% CI 2.86–25.61); whereas, bacteremia was inversely associated with all cause 30-day mortality (OR 0.33, 95% CI 0.15–0.71).

**Conclusions:**

In this cohort, bacteremia was not associated with a worse prognosis in elderly patients with pyelonephritis or urinary sepsis.

## Introduction

Urinary tract infection (UTI) is the most frequent bacterial infection in elders [1] and the second most common infectious disease for which elders are hospitalized [2], accounting for 10.3% of infectious disease hospitalization in the United States [3]. Moreover, UTI is the most frequent origin of community acquired bacteremia and sepsis in elderly patients [4, 5].

Bacteremia has been considered as a classic marker of severe disease [6, 7], but the evidence regarding its effects on clinical outcomes mainly come from studies with different sources of bacteremia, in which respiratory and abdominal sources have worse prognosis [8]. There are few studies on the effects of bacteremia on outcomes in UTI. Two previous studies on the clinical effect of bacteremia on outcomes in adults showed contradictory results [9, 10] and a retrospective study in elderly patients with UTI requiring hospitalization showed no effect of bacteremia, neither on hospital mortality nor length of hospital stay [11]. Furthermore, elderly patients admitted to hospital with UTI frequently have a high number of comorbid conditions that have an influence on outcomes [12]. Bacteremia has been found more frequently in very old people, making age itself another confounding factor in outcomes, since age has been linked with worse prognosis in UTI [7, 10, 12]. Therefore, there is a need for reliable data regarding the effect of bacteremia on outcomes in elderly patients with severe UTI admitted to hospital.

To address this issue, we performed a prospective study to compare outcomes in patients older than 65 years with pyelonephritis or urinary sepsis with and without bacteremia.

## Methods

### Study location and patients

This study was undertaken at 5 university and non-university Spanish hospitals, over a 10-month period from February 2016 to December 2016. All patients aged >65 years admitted to the Departments of Internal Medicine with a diagnosis of acute pyelonephritis or urinary sepsis, in which urine and blood cultures were obtained in the emergency departments were eligible for this research.

### Study design and data collection

A prospective multicentre observational cohort study design was used with the main outcome being all cause 30-day mortality. Secondary outcomes assessed were: Transfer to intensive care unit (ICU), length of hospital stay and hospital mortality. Outcomes were analysed according to whether blood cultures were positive or negative.

**Fig 1:**
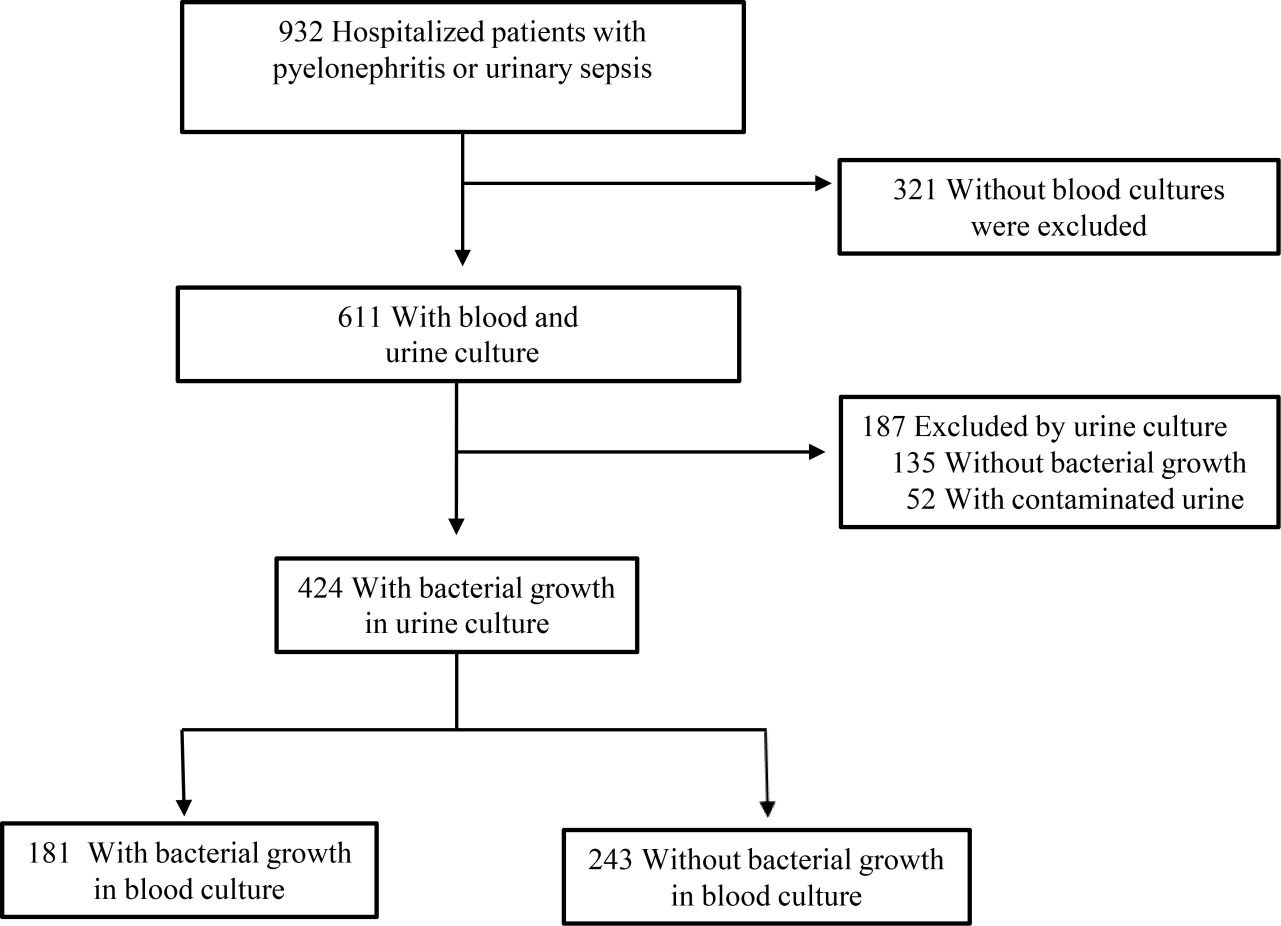
Flowchart for the selection of urinary tract infection cases.

Patients were considered for inclusion in the study if they had a diagnosis of acute pyelonephritis or urinary sepsis and urine culture was obtained at the emergency department before initiation of antimicrobial therapy. Patients were excluded if blood cultures were not collected or if urine culture revealed growth of >2 different bacterial species, which was considered to be contaminated, or lack of growth in urine culture [13]. The selection of patients for inclusion is shown in Fig 1.

Demographic, clinical and microbiological patient data were collected after the completion of the discharge report. The study design precludes that the researcher can influence the diagnostic tests requested or the treatment administered. The following characteristics were recorded from automated patient medical records: age; sex; comorbidities (diabetes mellitus, chronic obstructive pulmonary disease, previous stroke, dementia, solid neoplasia, and chronic kidney disease); severity of underlying conditions according to the McCabe classification [14]; health care-associated UTI (hospitalization for ≥2 days in the past 90 days; nursing home residence or previous antibiotic use in the past 90 days); recurrent urinary infection (≥3 episodes/year); urinary catheter; functional or anatomical abnormality of the urinary tract; any urologic intervention within 30 days; clinical variables present at admission and laboratory data to calculate APACHE II score and to make sure that the diagnosis of pyelonephritis, severe sepsis or septic shock were consistent with the study definitions; urine culture; blood culture; antibiotic treatment; inadequate empirical antibiotic treatment (IEAT); transfer to ICU, length of hospital stay; hospital mortality; and all cause 30-day mortality.

This study was approved by the Hospital Universitario Dr. Peset’s Clinical Investigation Ethics Committee and complies with ethical standards. Informed consent was waived as it was an observational study and data were anonymously analized.

### Definitions

Acute pyelonephritis was defined as the presence of two of the following: (a) axillary temperature ≥ 38.3°C or chills; (b) flank pain or costovertebral angle tenderness or pain on bimanual palpation of the kidney; and (c) mictional syndrome (including two or more of the following; dysuria, frequency, suprapubic pain or urgency), together with a positive urine culture [15]. Sepsis, severe sepsis and septic shock were defined following the criteria of the American College of Chest Physicians and Society of Critical Care Medicine Consensus Conference [16]. Positive urine culture results were defined as growth of >10^5^ cfu/mL of 1 or 2 pathogens. Bacteremia was defined as the growth of any pathogen in the blood culture. Isolation of coagulase-negative *Staphylococcus*, Viridans streptococci, or *Corynebacterium* in one of the pair of blood cultures were considered as a contaminant and these cases were included in the negative blood culture group. A discordant culture result was defined as a positive blood culture with a related urine culture that showed growth of another microorganism. The McCabe classification for underlying diseases includes 3 categories: 1. Non-fatal (death is not expected to occur in the next 5 years), 2. Ultimately fatal (death is expected to occur between 3 months and 5 years), and 3. Rapidly fatal (death is expected in the next 3 months). Inappropriate empirical antimicrobial therapy (IEAT) was considered as the occurrence of infection that was not effectively treated at the time when the causative microorganism and its antibiotic susceptibility were known [17, 18].

### Statistical analysis

We compared epidemiological and clinical distributions among patients with and without bacteremia using the chi-squared test for categorical variables and the Student’s t test for quantitative variables. The relationship between outcomes and bacteremia and risk factors for all cause 30-day mortality were analyzed by univariate logistic regression. Bacteremia and predictors for all cause 30-day mortality identified in the univariable analysis were included in a multivariable logistic regression model. SPSS v22.0 was used to perform the statistical analysis. All P values were two-tailed, and P values of 0.05 or less were considered to indicate statistical significance.

## Results

A total of 424 patients aged > 65 years with acute pyelonephritis or urinary sepsis with a positive urine culture and blood cultures taken at the emergency departments were included in the study. The average age of the total of the series was 79.9±7.9 years, with 54% of women. There were 102 (24.1%) with severe sepsis and 21 (5.0%) with septic shock. Fifteen (3.5%) patients were transferred to ICU. The average length of hospital stay was 9.3±6.2 days. The average APACHE II score was 20.2±7.8. All cause 30-day mortality was 11.6% and hospital mortality 5.0%.

The epidemiological and clinical characteristics of the patients on admission according to the presence of bacteremia are shown in Table 1.

**Table 1:** Baseline characteristics of the patients.

Characteristic	Bacteremic UTI N = 181 (42.7%)	Non-bacteremic UTI N = 243 (57.3%)	*P* Value
**Age, mean±SD**	80.7±7.4	79.4 ±8.4	0.070
**Female sex, no. (%)**	99 (54.7)	131 (53.9)	0.872
**Comorbidities, no. (%)**			
**Diabetes mellitus**	76 (42.0)	88(36.2)	0.227
**COPD**	30 (16.6)	33 (13.6)	0.391
**Previous stroke**	57 (31.7)	69 (28.5)	0.484
**Dementia**	62 (34.3)	77 (31.7)	0.578
**Solid neoplasia**	33 (18.3)	47 (19.3)	0.793
**Chronic kidney disease**	69 (38.1)	76 (31.3)	0.142
Mc Cabe’s classification ≥2*	122 (67.4)	139 (57.4)	0.037
**Health care-associated UTI, no. (%)**	131 (72.4)	172(70.8)	0.719
**Hospitalization for ≥ 2 days in the past 90 days**	89 (49.2)	125 (51.4)	0.644
**Nursing home residence**	65 (35.9)	57 (23.5)	0.005
**Previous antibiotic use in the past 90 days**	115 (63.5)	129 (53.1)	0.031
**Recurrent urinary infection (≥ 3 episodes/year)**	86 (47.8)	91 (37.6)	0.036
**Urinary catheter**	89 (49.2)	80 (32.9)	0.001
**Functional or anatomical abnormality of the urinary tract**	45 (25.0)	60 (24.8)	0.961
**Pyelonephritis**	34 (18.8)	51 (20.9)	0.575
**Severe sepsis**	52 (28.7)	50 (20.6)	0.052
**Septic shock**	10 (5.8)	11 (5.3)	0.825
**APACHE II score, mean ±DS**	23.6 ± 8.5	17.3 ± 5.7	<0.001

UTI = urinary tract infection; COPD = chronic obstructive pulmonary disease; APACHE II = Acute physiology and chronic health evaluation classification system. Results with significant differences are indicated in boldface

*Ultimately or rapidly fatal disease according to the McCabe classification

One hundred eighty-one (42.7%) patients had bacteremic UTI. These patients had higher percentages of ultimately or rapidly fatal disease according to the McCabe classification (67.4% vs. 57.4%; p = 0.037), came more frequently from nursing home residence (35.9% vs. 23.5%; p = 0.005), had higher percentages of previous antibiotic use in the past 90 days (63.5% vs. 53.1%, p = 0.031), recurrent urinary infections (47.8% vs. 37.6%, p = 0.036), urinary catheter (49.2% vs. 33.9%, p = 0.001) and higher APACHE II score (23.6±8.5 vs. 17.3±5.7, p<0.001).

The all cause 30-day mortality was not different in the bacteremic UTI group compared to the non-bacteremic group (9.4% vs. 13.2%, p = 0.223). Likewise, there were no differences in any of the other outcomes analyzed between bacteremic-UTI and non-bacteremic UTI (see Table 2).

**Table 2:** Relationship between outcomes and bacteremia.

	Bacteremic UTI N = 181 (42.7%)	Non-bacteremic UTI N = 243 (57.3%)	OR (95% CI)	*P* Value
**Transfer to ICU, no. (%)**	8 (4.4)	7 (2.9)	1.559 (0.555–4.381)	0.400
**Length of hospital stay, days, mean±SD**	9.7 ± 4.6	9.0 ± 7.3		0.252
**Hospital mortality, no. (%)**	6 (3.3)	15 (6.2)	0.521 (0.198–1.371)	0.187
All cause 30-day mortality, **no. (%)**	17 (9.4)	35 (13.2)	0.680 (0.365–1.268)	0.223

UTI, urinary tract infection; ICU, intensive care unit

The etiology of UTI were monomicrobial in 395 (93.2%) cases and polymicrobial in 29 (6.8%) cases, in which two microorganisms were isolated. The microorganisms isolated were: *Escherichia coli* 278 (65.6%), *Klebsiella pneumoniae* 36 (8.5%), *Pseudomonas aeruginosa* 29 (6.8%), *Enterococcus faecalis* 23 (5.4%), *Proteus* spp. 17 (4%) and others 41 (9.6%). Fifty-four (17.5%) cases were ESBL-producing *E*. *coli* and 14 (4.5%) cases were ESBL-producing *K*. *pneumoniae*. Two cases were carbapenemase-producing *K*. *pneumoniae*. Microorganisms isolated in urine and blood cultures were concordant in 406 (95.7%) cases. Discordant microorganisms isolated in blood cultures were the following: *E*. *coli* (n = 7), *K*. *pneumoniae* (n = 3), *Proteus* (n = 2), *Enterobacter cloacae* (n = 2) and others (n = 4). Inadequate empirical antimicrobial therapy (IEAT) was given in 111 cases (26.2%), but it was as high as 48.4% in cases caused by ESBL-producing enterobacteria. Both cases caused by carbapenemase-producing *Klebsiella pneumonaie* received IEAT.

Risk factors for all cause 30-day mortality are shown in Table 3. Bacteremic UTI was not associated with all cause 30-day mortality in the univariate analysis, but it was inversely associated with all cause 30-day mortality by multivariate analysis (OR 0.331, 95% CI 0.154–0.710, p = 0.005). Age (OR 1.052, 95% CI 1.001–1.105, p = 0.045), ultimately or rapidly fatal disease (OR 10.475, 95% CI 2.962–37.046, p<0.001) and septic shock (OR 8.565, 95% CI 2.864–25.611, p<0.001) were associated with all cause 30-day mortality.

**Table 3:** Risk factors for all cause 30-day mortality.

	Univariate analysis			Multivariate analysis
Variables	SURVIVAL N = 375 (88.4%)	DEATH N = 49 (11.6%)	*P* Value	OR (95% CI); *P* Value
**Bacteremic UTI, no. (%)**	164 (43.7)	17 (34.7)	0.229	0.331 (0.154–0.710); **0.005**
**Age, mean±SD**	79.46±7.93	83.71±7.34	**<0.001**	1.052 (1.001–1.105); **0.045**
McCabe’s classification ≥ 2*, no. (%)	215 (57.5)	46 (93.9)	**<0.001**	10.475 (2.962–37.046); **<0.001**
**Severe sepsis, no. (%)**	82 (21.9)	20 (40.8)	**0.004**	2.034 (0.954–4.337): 0.066
**Septic shock, no. (%)**	11 (3.2)	10 (23.3)	**<0.001**	8.565 (2.864–25.611); **<0.001**
**APACHE II, mean±SD**	19.93±7.65	22.32±8.67	0.093	---
**IEAT, no. (%)**	97 (26.0)	14 (30.4)	0.521	---

UTI = urinary tract infection; APACHE II = Acute physiology and chronic health evaluation classification system; IEAT = inadequate

empirical antibiotic treatment

*Ultimately or rapidly fatal disease according to the McCabe classification

## Discussion

Our results indicate that prognosis in elderly patients with bacteremic acute pyelonephritis or urinary sepsis is not worse than in those without bacteremia. These findings consolidate the results of two previous works, in adults [9] and elderly [11], which also indicated that bacteremia is not a risk factor for mortality in elderly patients with severe UTI requiring hospitalization.

Contrary to what might have been expected from studies on severe sepsis and septic shock, including patients with various sources of infection, an inverse relationship was found between bacteremia and all cause 30-day mortality. Furthermore, none of the other outcomes evaluated, i.e. transfer to ICU, length of hospital stay and hospital mortality was associated with bacteremia. These facts showed a consistent lack of harmful effect of bacteremia on prognosis in elderly patients with UTI. Moreover, both hospital mortality and all cause 30-day mortality were lower in patients with bacteremia than in those without bacteremia (3.3% vs. 6.2%, p = 0.187 and 9.4%, vs. 13.2%, p = 0.223; respectively), although the difference was only statistically significant in all cause 30-day mortality. A possible reason for the relatively benign course for invasive disease in UTI is that the urinary system is largely self-draining, making it easier to achieve source control. These low rates of mortality in our study are not surprising, since the high rates of mortality in sepsis and septic shock come from studies in which urinary infections were only a small percentage of the total of the series [19] and it is well known that urinary source of sepsis has a lower risk of death [20].

As in previous works [21], in this study older age was associated with a slight increase in mortality. Older patients are often nutritionally or immunologically impaired, making them an easy target for infection and its associated complications [22]. The severity of underlying disease, according to the McCabe classification, was also found to be a predictor of death, measured as all cause 30-day mortality. This result is in line with previous studies that showed ultimately or rapidly fatal disease according to the McCabe classification was associated with mortality in severe sepsis, including urinary sepsis [23], and in community-acquired UTI requiring hospitalization [11]. Septic shock was also found to be a predictor of mortality, in accordance with other studies that showed septic shock to be a risk factor for mortality in patients admitted to hospital with urinary and other sources of severe infection [11, 15].

However, by multivariate analysis bacteremia was inversely associated with all cause 30-day mortality in our study. This association should be viewed as exploratory, given the small number of outcomes. Although bacteremia did not suggest a worse prognosis we acknowledge that taking blood cultures in elderly patients admitted to hospital with severe UTI could be clinically useful in some cases, such as those in which urine is contaminated or the urine culture does not grow any microorganism and blood cultures could be the only way of knowing the etiology.

This study has several limitations. First, we had to rely on the symptoms recorded by the attendant physicians in the medical records. Therefore, misclassification of urinary sepsis in some cases of sepsis from other sources with asymptomatic bacteriuria could not be completely assured. However, the high concordance in bacteria isolated from urine and blood suggest a high degree of accuracy of urinary sepsis diagnosis. Second, mortality was quite low, especially hospital mortality, making it hard to evaluate predictors of mortality. Consequently, additional studies are needed to determine risk factors for in-hospital mortality. Third, we do not know whether the all cause 30-day mortality was attributable to infection, therefore mortality in some cases may not have been related to infection, especially taking into consideration that the patients were very old and had comorbidities. Fourth, although delay in the initiation of appropriate antibiotic therapy has been recognized as a risk factor for mortality [24], we were not able to determine the influence of timing of the antibiotic administration.

In conclusion, among elderly patients with pyelonephritis or urinary sepsis admitted to hospital, bacteremia is not associated with a worse clinical outcome. Patients’ comorbidity and septic shock were predictors for all cause 30-day mortality.
